# Bacteriology

**Published:** 1904-12-24

**Authors:** 


### BACTERIOLOGY.

**The Rheumatic Diseases.**—Edsall and Larensen [1] writing concerning the nature of certain cases of chronic polyarthritis, maintain that many described as chronic rheumatism and arthritis deformans are really tubercular in origin. They quote Poncet's experiments, in which fluid obtained from so-called chronic rheumatic joints were found to excite tuberculosis when injected into guinea-pigs. The writers base their assumption on the results observed to follow the injection of tuberculin. Thus of 18 cases of chronic rheumatism or rheumatoid arthritis eight gave no reaction, three gave a doubtful reaction, and seven gave a positive result. The most conclusive case was that of a child suffering from Still's disease, in whom tuberculin gave rise to a marked local and general reaction, and in whose lymph glands, which had been removed by operation, there were found acid fast bacilli. Unfortunately these bacilli were not pathogenic to guinea-pigs. In all but one of the eight positive cases there was marked enlargement of the lymph glands. Lewis and Longcope [2] record a case of diplococcus rheumaticus infection in a girl aged eight, who had suffered from acute rheumatism, chorea, hyperpyrexia, and endocarditis. Blood drawn from a vein was inoculated into milk, and subcultures of Poynton and Payne's diplococcus were subsequently made in broth and agar. Milk was acidified, and from this formic acid was obtained by distillation. A series of rabbits were inoculated, and after an incubation period of from four to nine days, joint lesions were demonstrable, and the diplo-streptococcus was obtained from the joint fluids. Myocarditis, fungating endocarditis, and infarction of the spleen and kidneys also resulted in some cases, whilst in others subcutaneous nodules appeared on the extensor tendons. The evidence of the specific nature of this organism and of its differentiation from the streptococcus pyogenes was not evident to the writers. Beattie [3] has made a study of the cultural and other characteristics of the diplococcus rheumaticus. It is smaller than the ordinary streptococcus, never forms long chains, is apt in old cultures to show degeneration forms, and is rather oval in shape. It is stained by Gram's method. It forms a sediment in alkaline broth, producing acid but not indol. This formation of acid is most marked in peptone solution, least so in glycerine bouillon. In gelatine growth is evident along the needle track in two days, but the medium is never liquefied. Agar cultures grow better at 20° C. than at 37° C., the colonies being semi-transparent without tendency to spread. On blood-agar the diplococcus flourishes, turning the blood a rusty-brown or chocolate colour, and retaining vitality for many months. Growth is also free on blood serum. Milk is coagulated and acidified in three days. Anærobic media do not give a favourable growth. Intravenous injection into animals give rise to endocarditis, polyarthritis, and chorea. From the foregoing it will be seen that the micrococcus rheumaticus differs from a streptococcus in that the former grows more abundantly and readily on gelatine and grows on agar better at 20° C. than at 37° C.; it has considerable acid-forming powers and a high vitality on ordinary media. Unlike the streptococcus it does not cause suppurative lesions.

**A New Pathogenic Throat Organism.**—Stone [4] records an epidemic of angina, accompanied by profound toxæmia which occurred in the state of Vermont. The tonsils were swollen, the soft palate and uvula œdematous and white tenacious false membrane covered the fauces. The micro-organism exciting this condition was, in his opinion, a diplococcus having the following characteristics: The two parts of the diplococcus were of unequal size, the adjacent sides were flattened. Stained with Loeffler's blue, a distinct metachromatic granule was seen. The diplococci, when examined from cultures, are seen to be clumped in irregular bunches held together by gelatinous material. They vary in diameter from $\cdot 5\mu$ to $1\mu$. They are stained by Gram's method. Growth on agar, and blood serum is in the form of white, raised, tenacious colonies at first discrete, regular, and pulvinate, but later coalescing into a continuous nodular line. In bouillon it forms a stringy, white sediment. The growth of the diplococcus is not accompanied by the production of acid, gas, or indol. It requires oxygen, and the thermal death point is between 60° and 70° C. The organism is variably pathogenic for laboratory animals, sometimes proving fatal in 12 hours. The poison could not be isolated from a bouillon culture by Breiger's method, and is therefore a true toxin and not a ptomaine.

**Small-pox and Vaccinia.**—Korté [5] describes an amœboid protozoon which is, he states, constantly associated with small-pox and vaccinia. It measures about 1-2,500th of an inch, and has the form of an oblate spheroid containing intracellular spores. In stored lymph the mobility of the parasite is lost. In vaccination the amœba is to be found in the papule on or about the ninth day. The organism can only be demonstrated in hanging drop preparations of fresh lymph after centrifugalisation, and must be stained by suspending them in normal saline solution tinted with Loeffler's blue. Permanent preparations are unsatisfactory, as the organism is broken by the slightest pressure. If, however, a film be spread with great care, fixed in formalin or alcohol and ether, stained with methylene blue, and mounted in equal parts of glycerine and water in a shallow cell fairly good results are sometimes obtained.

**Bacteria in Inflammatory Diseases of the Alimentary Canal.**—Hewetson[6] found in 44 cases of non-suppurative appendicitis that the appendix was stenosed in 25. In 14 of these the distal chamber was found to contain micro-organisms—B. coli, a torula, staphylococcus p. aureus and a streptobacillus; 11 were sterile. Only 1 of the 19 non-stenosed cases was sterile, in the others the organisms present were B. coli, staphylococci and streptococci. In suppurative appendicitis it was found that most of the micro-organisms could be isolated by ærobic cultures, the anærobic preparations being of little additional assistance. The organisms present in suppurative appendicitis were B. coli in 34 cases; micrococci in 18; streptococcus pyogenes in 7; diplococci in 5; B. pyocyaneus in 5; pneumococci in 2; diplobacilli in 1; actinomycosis in 7. Cultures were made from the human œsophagus, stomach and small intestine at times when such viscera had been opened for operative purposes. The stomach contained few germs if healthy, but in cases of ulceration the number of micro-organisms was considerably increased. Exactly 50 per cent. of the stomachs examined were sterile, the remainder showed torulæ, staphylo- and streptococci, B. coli, B. subtilis, sarcinæ, B. proteus and micrococci. Sixteen out of twenty-nine cultures taken from the jejunum were sterile, and such organisms as were found in stomach or jejunum were non-pathogenic. Ordinary pyogenic organisms are killed in the stomach in from 30 to 45 minutes, and bacilli die in from 60 to 90 minutes. The peritoneal effusion in ten cases of perforated gastric ulcer was examined and found to be sterile in four instances.

[1] Amer. Jour. of Med. Sci., Dec. 1903. [2] Ibid, Oct. 1904. [3] Brit. Med. Jour., Dec. 3, 1904. [4] Med. Rec., Aug. 13, 1904. [5] Brit. Med. Jour., Nov. 19, 1904. [6] Ibid., Nov. 26, 1904.

